# Chemical and sensory analyses of cultivated pork fat tissue as a flavor enhancer for meat alternatives

**DOI:** 10.1038/s41598-024-68247-4

**Published:** 2024-07-31

**Authors:** Emily T. Lew, John S. K. Yuen, Kevin L. Zhang, Katherine Fuller, Scott C. Frost, David L. Kaplan

**Affiliations:** 1 Tufts University School of Engineering, Medford, MA 02155 USA; 2 Tufts University School of Arts and Sciences, Medford, MA 02155 USA; 3 Tufts University Friedman School of Nutrition, Boston, MA 02111 USA

**Keywords:** Biological techniques, Cell biology

## Abstract

The emerging field of cellular agriculture has accelerated the development of cell-cultivated adipose tissue as an additive to enhance the flavor of alternative meat products. However, there has been limited research to evaluate the sensory profile of in vitro-grown tissues compared to conventionally obtained animal fat. This study aimed to investigate the aromatic characteristics of cell-cultivated fat tissue as a flavor enhancer for meat alternatives. Porcine dedifferentiated fat (PDFAT) cells were clonally isolated and differentiated into adipocytes. This cultured adipose tissue was then analyzed alongside native porcine fat using gas chromatography-mass spectrometry (GC/MS) coupled with descriptive sensory analysis by human consumers. This evaluation enabled quantitative and qualitative assessments of volatile compounds released during cooking for both in vitro and in vivo porcine fats. The volatile profiles generated during the cooking process and fatty aroma characteristics reported by sensory consumers were largely similar between the two fat sources, with some differences in select compounds and aroma attributes. Ultimately, the consumers found comparable overall liking scores reported between the conventional and cultured porcine fats. These findings provide valuable sensory evidence supporting the viability of cell-cultivated adipose tissue as a flavor component of meat alternatives, substituting for conventional animal fat.

## Introduction

The increasing global population and the environmental, ethical, and health-related concerns associated with traditional livestock farming have accelerated the search for sustainable and humane alternatives to industrialized meat production^1–6^. Currently, plant-based meats made from soy, tofu, and textured vegetable proteins serve as alternative protein sources for vegetarians, vegans and other consumers interested in reducing their meat consumption. Similarly to cultivated meat, plant-based proteins have a much lower environmental impact compared to animal-derived proteins due to lower water use, land use and reduced greenhouse gas emissions^7^. Although many consumers are interested in plant-based meats, taste is a primary barrier to consumption due to flavor differences compared to conventional animal proteins^8^. In response to these challenges, recent advances in cellular agriculture have demonstrated the potential for producing cell-cultured adipose tissue, or fat, as a key component in replicating the sensory qualities and nutritional value of meat products^9–12^. However, while the theoretical advantages of cell-cultivated fat are widely discussed, limited empirical evidence exists and there remains a lack of comprehensive evaluations supporting the sensory characteristics and overall viability of cultivated fat as a meat alternative for traditional livestock-generated fat^13,14^.

Despite the limited presence in the consumer marketplace, many studies have been conducted to examine the potential for consumer acceptance of cultivated meat^15–17^. In these studies, sensory characteristics similar to conventional meat is one of the primary determinants of consumer acceptance^18,19^. Additionally, several reviews have been published which outline the hypothetical acceptance of cultivated meat, but do not discuss measurable sensory qualities of cultivated meat aroma or flavor^13,20–22^. Two sensory evaluation studies including real cultivated meat samples have been conducted which determined that the cultivated meat products closely resembled conventional analogues^23,24^. While these studies indicate that cultivated meat has the potential to emulate conventional animal products, gaps in these studies include using low numbers of human consumers and comparisons of cultivated meat to nonequivalent conventional products. To address these gaps, we present a study with over 50 consumers using a comparable target conventional meat product to evaluate the sensorial qualities of cultivated porcine adipose.

In our previous research, adipocytes were grown at a laboratory scale and mechanically aggregated into macroscale tissues resembling native pig fat^25^. These in vitro-grown porcine adipocyte tissues demonstrated similar fatty acid composition to native pig fat, suggesting the potential similarity in taste and aroma characteristics with native porcine adipose. Using lipidomic analysis, it was found that in vitro-grown and native porcine fats contained similar fatty acid profiles, especially intracellular triacylglyceride fractions. Significant differences were found in the phospholipid fraction where cultivated porcine fat contained lower amounts of 18:2 and 18:3 fatty acids and higher amount of 20:1. Media supplementation with Intralipid remedied this difference, whereby an increase of 18:2 and 18:3, and a decrease of 20:1 was found. Additionally, in vitro-grown and native porcine fats shared similar ratios of monounsaturated fatty acids, polyunsaturated fatty acids and saturated fatty acids with the Intralipid supplementation.

Building upon these previous findings, the goal of the present study was to evaluate the composition and sensory characteristics of cell-cultivated porcine adipose tissue, towards the evaluation of this process for flavor-enhancing components in alternative meat products. To achieve this objective, porcine fat was generated in vitro using a porcine dedifferentiated fat (PDFAT) cell subclone produced via single cell seeding from the original mixed population of primary DFAT cells. Gas chromatography-mass spectrometry (GC/MS) paired with human sensory evaluation were utilized to quantitatively and qualitatively assess volatile compounds released from both the in vitro and in vivo porcine fats during heating (cooked fat aroma). Additional GC/MS analyses using an olfactory detection port (ODP) was utilized to identify specific compounds responsible for various porcine fat characteristics reported during the human sensory trials.

## Results

### Study design

**Figure 1:**
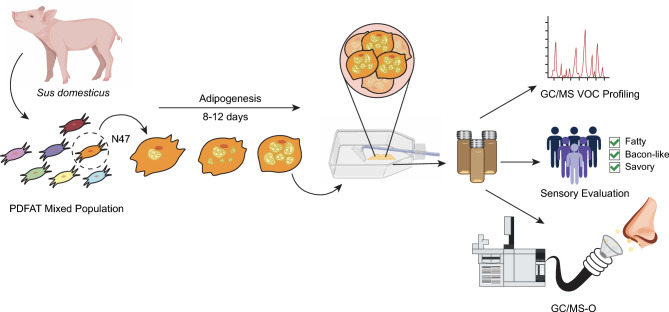
Graphical overview of the methodology. A clonal population of porcine dedifferentiated fat cells were isolated, mechanically aggregated via cell scraping and used for GC/MS and sensory analysis. The figure was created in Adobe illustrator by the first author.

Figure 1 shows an overview of the study design including isolation of a clonal population of PDFAT, macroscale aggregation of cultivated fat and use of fat samples in sensory workflow (Fig. 1).

### Clonally isolated PDFAT cells optimized for lipid accumulation were aggregated into 3D fat samples

**Figure 2:**
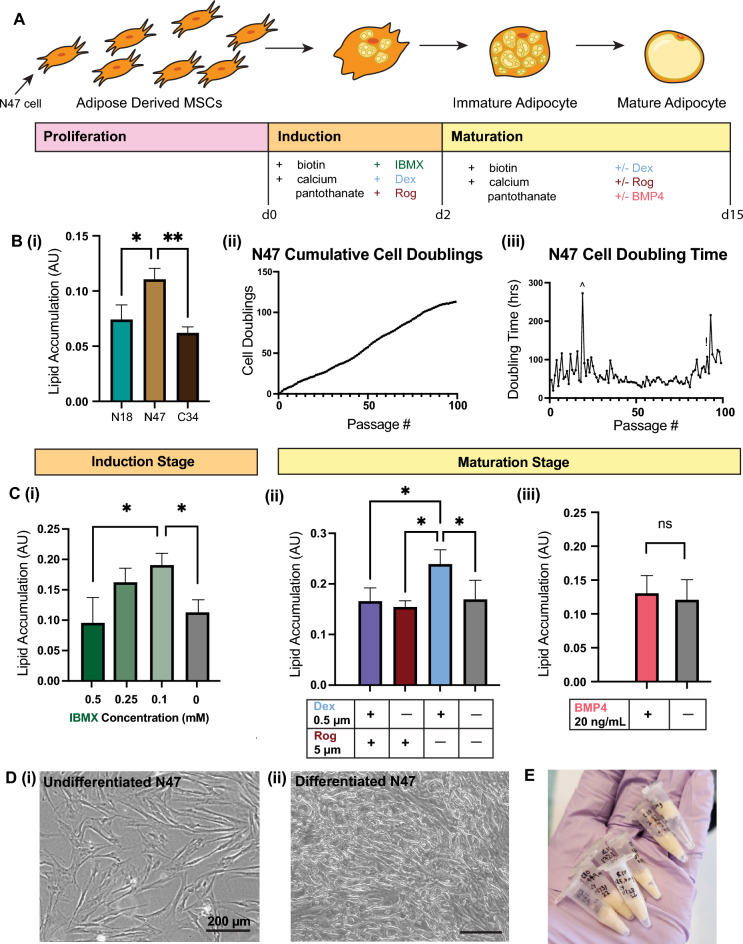
Single-cell PDFAT clones were grown and optimized for cultured fat. (**A**) Schematic depicting stages of adipogenesis and media components targeted for optimization. (**B**) Three porcine DFAT clones (P12) screened for lipid accumulation after eight days of adipogenesis via Oil Red O staining. (**B, i**) Screening of clones N18, N47 and C34. (**B, ii**) Cumulative cell doublings. Data was analyzed with one-way ANOVA, where p ≤ 0.05 (*) and p ≤ 0.01 (**) (**B, iii**) Hours per doubling. (^) indicates passage with low cell count due to poor detachment and (!) indicates passage 95 reporting no growth (data point not shown). (**C**) Media optimization for adipogenic induction and maturation stages. N47 was screened for lipid accumulation after eight days of adipogenesis via Oil Red O staining. (**C, i)** Presence of IBMX in the first 2–3 days of adipogenesis (P12, n = 3). Data was analyzed with one-way ANOVA, where p ≤ 0.05 (*). (**C, ii**) Presence of dexamethasone and rosiglitazone in lipid accumulation media (P3). Both dexamethasone and rosiglitazone were present for the first 2–3 days of adipogenesis (induction media). Data was analyzed with two-way ANOVA, where p ≤ 0.05 (*). (**C, iii**) Presence of BMP-4 at 20 ng/mL in the lipid accumulation stage (n = 3). Data was analyzed with unpaired t-test, where p ≤ 0.05 (*). (**D**) N47 morphology before and after differentiation. (**D, i**) Undifferentiated and (**D, ii**) differentiated cells after eight days of adipogenesis. Scale bar 200 um. (**E**) N47 adipocytes were mechanically harvested with a cell scraper and aggregated into masses of cultivated fat. Fat was aliquoted into portions of 150–200 mg in 0.6 ml Eppendorf tubes. The picture of fat in this figure was taken by the first author.

To obtain a pure cell line from previously isolated PDFAT, single cell sorting was used to separate single cells. The goal was to select a high preforming clone and optimize adipogenesis for a variety of differentiation media components (Fig. 2A). A total of 576 single cells were seeded into regular culture media (N), 50% conditioned media (C) and vitronectin supplemented media (V) in 96-well plates. At 24 h after seeding, wells were screened for successful single cell seeding indicated by isolated cell populations. Clonal populations were grown to confluence and visually screened for proliferation. Intracellular lipid accumulation of all viable clones was quantified with Oil Red O. Five clones N18, N20, N47, C15, and C34 were selected as they were found in the top 15 clones of the lipid accumulation screen (Supplementary Fig. S1) while previously exhibiting strong proliferation based on visual observation (data not shown). These five clones were monitored for six passages before reducing the screen to clones N18, N47, and C34. Clones N18, N47 and C34 were selected from the top five clones based on visual proliferation screening and lipid accumulation (data not shown). Clones N18, N47 and C34 were screened for proliferation through passage nine (Supplementary Fig. S2). Due to higher lipid accumulation, long-term culture continued with clone N47 (Fig. 2B, i). Across 100 passages, clone N47 had an average doubling time of 60.6 h. with an average of 1.15 doublings per passage (Fig. 2B; ii, iii). Media optimization was conducted for adipogenesis agonists dexamethasone, rosiglitazone, 3-isobutyl-1-methylxanthine (IBMX), and BMP-4 to better inform media formulations for the mixed population PDFAT and clone N47. Standard adipogenic culture media contained both dexamethasone and rosiglitazone in adipogenic induction and lipid accumulation media and 0.5 mM IBMX. Culture media was altered to omit rosiglitazone in lipid accumulation media and reduce IBMX concentration to 0.1 mM thereafter (Fig. 2C; i, ii). While a high degree of lipid accumulation was observed, lipid morphology remained multilocular with these optimizations (Supplementary Fig. S3). Additionally, the presence of the differentiation factor BMP-4 was tested at 20 ng/mL but did not yield higher lipid accumulation (Fig. 2C, iii). Cells became lipid-laden within eight days of adipogenesis (Fig. 2D). Approximately 1 g of adipose tissue was harvested via cell scraping for subsequent downstream experiments (Fig. 2E). With an initial seeding density of 11,000 cells/cm^2^, T175 flasks became confluent within approximately 4 days. One culture flask with 175 cm^2^ surface area yielded approximately 150 mg of cell mass after eight days of adipogenesis. After harvest, scraped adipocytes were stored at − 80 °C for GC/MS and sensory analysis.

### Volatile chemistry of cooked porcine fat was analyzed using GC/MS

**Figure 3:**
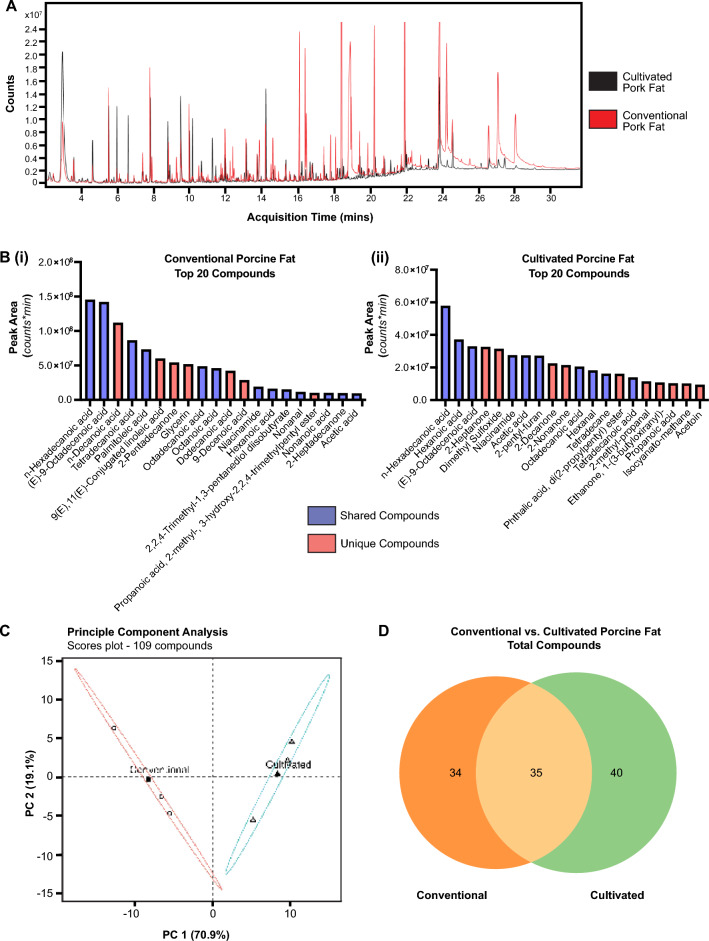
Volatile organic compound (VOC) profiling of conventional (livestock sourced) and cell cultivated porcine fat. (**A**) Total ion chromatograms of conventional pork belly fat (red) and N47 cultured porcine fat (black). Volatile and semi-volatile chemistry from each fat sampled using DHS with subsequent analysis using gas chromatography and mass spectrometry detection. Samples were heated to 120 °C with 15 min incubation and 30 min trapping time. (**B**) Comparison of top 20 volatile compounds from (**B, i**) conventional pork fat and (**B, ii**) N47 cultivated porcine fat using peak area (n = 3). Red denotes compounds unique to either conventional or cultivated fat samples. Blue denotes compounds that are shared between conventional and cultivated fat samples within total compounds. (**C**) Principal component analysis showing the relationship of the conventional and cultivated fat samples as defined by the measured volatile chemistry (n = 3). The open circle (ο) indicates the conventional fat sample replicates, and the open triangle (∆) indicates the cultivated fat sample replicates. The closed shape denotes the projection of the average. (**D**) Venn diagram showing the commonality of identified compounds. Shared compounds are indicated in the overlapping area of each oval.

GC/MS analysis of the conventional and in vitro fat samples revealed similar volatile profiles, apart from additional low volatility (high retention) compounds found in the conventional fat sample and additional high volatility (low retention) compounds found in the in vitro fat sample (Fig. 3A). Compounds identified by GC/MS in cultivated and conventional porcine fat were ranked by peak area. Comparison of the compounds present in cultivated and conventional porcine fat revealed that they share long and medium chain fatty acid compounds such as hexadecenoic acid, hexanoic acid, (E)-9-octadecanoic acid, octadecanoic acid, tetradecanoic acid, among others (Fig. 3B, Table 1, Table 2, Supplementary Table S1). In total, between the two fat samples 109 compounds were returned. Principal component analysis (PCA) was applied to this data to evaluate the relationship between the fat samples. PCA results (Fig. 3C) captured 90.0% of the variation, with the first component accounting for 71.9%. Within the scores plot, two clusters are shown with correspond to a fat sample; the cultivated sample is positioned along the positive direction of PC 1 and the conventional sample is positioned along the negative direction. The analytical replicated for each sample are clusters by sample along the second component, with the sample mean projected into the middle of each cluster. Compounds were not equally distributed between the two fat samples. Figure 3D displays a Venn Diagram illustrating the differences and commonality of compounds between the two fat samples. Between the two samples, 35 compounds were identified as common. The complete list of 35 shared compounds identified in cultivated and conventional porcine fat can be found as Supplementary Table S1 online. Unique compounds identified in each fat sample can be found as Supplementary Table S2 and Supplementary Table S3 online.

**Table 1:** Top 20 volatiles by peak area from conventional porcine fat.

Compound	Peak area	Retention time (min.)	RI (meas.)	RI (ref.)	References
n-Hexadecanoic acid	145,163,036	23.9	2908	2910	^78^
(E)-9-Octadecenoic acid	142,004,120	27.1	3066	3173	^79^
*n-Decanoic acid	111,782,473	18.5	2272	2279	^80^
Tetradecanoic acid	86,431,341	22.0	2696	2712	^81^
Palmitoleic acid	73,204,134	24.3	2944	2960	^79^
9(E),11(E)-Conjugated linoleic acid	59,967,809	28.1	3234	3168	^82^
*2-Pentadecanone	54,228,020	16.1	2027	2011	^83^
Glycerin	51,668,188	18.9	2316	2322	^84^
Octadecanoic acid	48,567,818	26.6	3092	3181	^85^
Octanoic acid	45,795,310	16.4	2058	2070	^86^
Dodecanoic acid	42,205,675	20.3	2483	2502	^78^
9-Decenoic acid	28,614,654	19.0	2332	2348	^87^
Niacinamide	19,010,696	24.6	2971	2960	^79^
*Hexanoic acid	16,083,840	14.2	1844	1849	^80^
2,2,4-Trimethyl-1,3-pentanediol diisobutyrate	15,084,606	14.7	1887	1591	^88^
*Nonanal	11,334,456	8.8	1395	1390	^89^
Propanoic acid, 2-methyl-, 3-hydroxy-2,2,4-trimethylpentyl ester	9,972,895	14.5	1873	**	**
Nonanoic acid	9,881,773	17.5	2165	2169	^86^
2-Heptadecanone	9,581,884	18.1	2237	2255	^79^
*Acetic acid	9,404,231	10.4	1518	1465	^90^

“*” Denotes compounds in the top 20 that were also detected with the GC/MS olfactory detection port. Tentative identification is indicated by “**” (n = 3).

**Table 2:** Top 20 volatiles by peak area from cultivated porcine fat.

Compound	Peak area	Retention time (min.)	RI (meas.)	RI (ref.)	Reference
n-Hexadecanoic acid	57,742,859	23.9	2907	2910	^78^
*Hexanoic acid	37,043,963	14.2	1844	1849	^80^
(E)-9-Octadecenoic acid	32,767,970	22.4	2741	3173	^79^
2-Heptanone	32,461,734	19.5	2394	1184	^86^
*Dimethyl sulfoxide	31,348,131	3.1	864	1582	^91^
Niacinamide	27,397,941	24.6	2972	2960	^79^
*Acetic acid	27,279,476	10.4	1517	1465	^90^
Furan, 2-pentyl-	27,107,789	6.6	1234	1231	^89^
2-Decanone	22,348,937	17.6	2178	1493	^89^
2-Nonanone	21,354,871	6.0	1189	1386	^89^
Octadecanoic acid	20,376,824	26.6	3092	3181	^85^
*Hexanal	18,069,051	4.7	1093	1078	^92^
Tetradecane	16,025,271	8.9	1400	**	**
Phthalic acid, di(2-propylpentyl) ester	15,974,451	27.5	3175	**	**
Tetradecanoic acid	13,778,200	22.0	2695	2712	^81^
2-methyl-Propanal	11,318,251	2.3	804	812	^86^
1-(3-butyloxiranyl)-Ethanone	10,646,196	11.9	1643	**	**
Propanoic acid	10,145,153	10.7	1537	1525	^93^
Methane, isocyanato-	10,031,276	2.9	850	**	**
Acetoin	9,292,528	10.4	1517	**	**

“*” denotes compounds in the top 20 that were also detected with the GC/MS olfactory detection port. Tentative identification is indicated by “**” (n = 3).

### Consumers were able to discriminate between conventional and cultivated fat samples

Significant discrimination was shown between the two fat samples. 41 out of 54 consumers were able to correctly discriminate between the conventional (livestock sourced) and cultivated fat tissue. Consumers were presented with three fat samples in random combinations containing either two cultivated and one conventional sample or one cultivated and two conventional samples. Despite the different sample presentations, enough consumers were able to correctly discriminate between the cultivated and conventional samples to demonstrate statistical significance. The critical number of correct responses required to demonstrate statistical significance (p < 0.05) in a triangle test is 25/54^26^ (Supplementary Table S4). The full summary of triangle test responses can be found in Supplementary Table S5.

### Meat-like aroma attributes were largely similar for conventional and cultivated fats, while certain attributes were slightly more prevalent in cultivated fat samples

**Figure 4:**
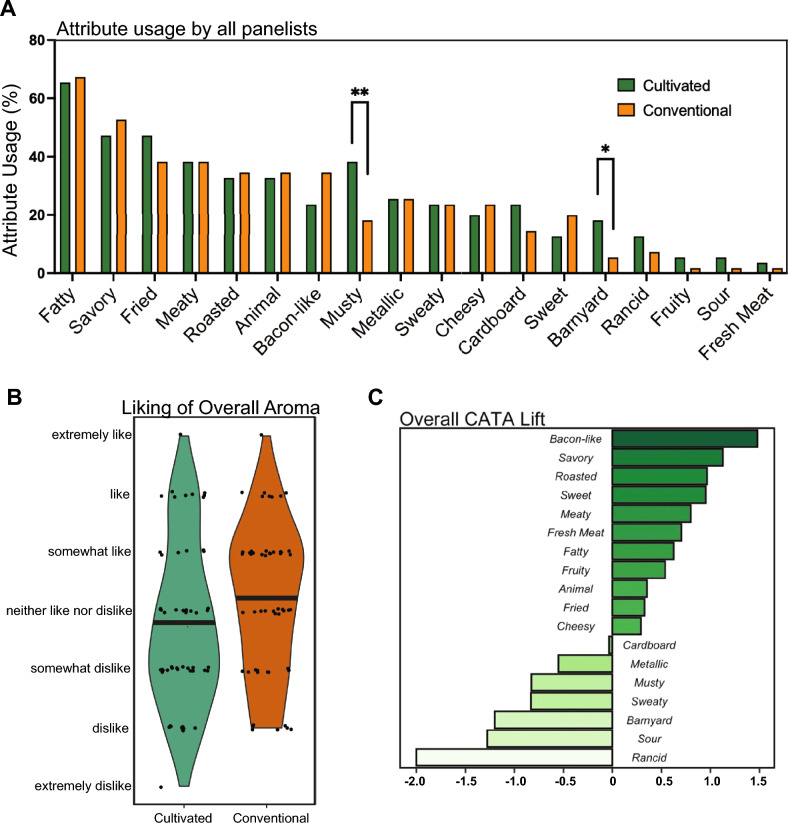
Aroma evaluation conducted with 55 consumers. (**A**) CATA results showing overall attribute usage by all participants for the cultivated and conventional porcine fat samples. Sample groups were compared with Fischer’s exact test, where p ≤ 0.05 (*) and p ≤ 0.01 (**). (**B**) Violin plot demonstrating distribution of overall liking of aroma between cultivated and conventional porcine fat samples on a 7-point hedonic scale of Extremely Dislike to Extremely Like. Mean values for each fat sample are shown by a solid black line. (**C**) Penalty/lift analysis relating degree of liking to specific sensory attributes in cultivated and conventional porcine fat samples.

Consumers indicated their sensory perceptions of in vivo and in vitro-grown pig fat through a “check-all-that-apply” (CATA) task. An attribute list was generated using 18 common descriptors used in previously published sensory evaluations of meat products and aroma/flavor wheels^27–30^. Overall consumer usage of the 18 attributes is shown as a bar plot (Fig. 4A). The total height of each bar corresponds to the total usage percentage of each descriptor. For example, the term “savory'' was used 50% of the time by all consumers when evaluating cultivated pork fat. For both fat samples, the attribute “fatty” was checked most of the time followed by “savory'', “fried”, and “meaty” which were used 47.3%, 47.3%, and 38.2% of the time for cultivated fat samples. Term usage differed more greatly with negative attributes such as “musty” and “barnyard”, where consumers checked these attributes more often when evaluating cultivated fat samples.

To investigate the overall opinion of the aromas of the fat samples, consumers rated the overall aroma of the samples on a 7-point hedonic scale from “Extremely Dislike” to “Extremely Like” (Fig. 4B). The violin plot displays hedonic ratings for each fat sample. On average, consumers expressed no difference in preference with respect to the aroma of  the conventional or cultivated pig fat.

CATA-lift analysis was used to investigate the changes in the hedonic ratings when a specific attribute was detected compared with not detected (Fig. 4C)^31^. Penalty/lift analysis shows the mean hedonic difference for each of the 18 attributes. For example, when the attribute “bacon-like” was checked for either cultured or conventional fat samples, a 1.5-point mean increase in overall liking was found. Liking increased when consumers detected “bacon-like”, “savory”, “roasted”, “sweet”, “meaty”, “fatty”, “fruity”, “animal”, “fried” and “cheesy” aromas, while liking decreased when consumers detected “cardboard”, “metallic”, “musty”, “sweaty”, “barnyard”, “sour” and “rancid” aromas.

### Major contributors to “musty” and “barnyard” aromas in both conventional and in vitro-grown fat samples include medium-chain saturated fatty acids

Out of 69 compounds detected in conventional pork fat, 31 were selected as aroma-active (Table 3). Sensory impactful compounds were of similar classification with the addition of ketones and benzene derivatives. Out of 75 compounds detected in cultivated pork fat, 25 compounds were selected as aroma-active (Table 4). These compounds include aldehydes, alcohols, acids, sulfur-containing compounds, and furans.

**Table 3:** GC/MS-O analysis of conventional pork fat.

Compound	Aroma	RI (meas.)	RI (ref.)	References
3-methyl-Butanal	Butter	940	876	^84^
Pentanal	Sweet	975	979	^86^
Hexanal	Grass	1048	1078	^92^
12,15-Octadecadienoic acid, methyl ester	Sour	1095	**	**
Heptanal	Sweet	1144	1181	^92^
1-Pentanol	Dried hay	1222	1255	^86^
Octanal	Fatty	1256	1291	^86^
(Z)-2-Heptenal	Cooked fat	1298	1319	^94^
Nonanal	Fatty, grease	1381	1390	^89^
3,5-Octadien-2-ol	Roasted vegetables	1398	**	**
1,3-bis(1,1-dimethylethyl)-Benzene	Oil	1415	1426	^95^
(E) 2-Octenal	Rancid oil	1423	1434	^86^
1-Octen-3-ol	Grassy	1449	1456	^86^
Acetic Acid	Vinegar	1461	1465	^90^
Decanal	Rancid	1501	1485	^96^
Benzaldehyde	Nuts	1533	1508	^78^
(E)-2-Nonenal	Fried fat	1541	1542	^86^
(E)-2-Decenal	Fatty	1580	**	**
d-Mannose	Stale	1662	**	**
Benzeneacetaldehyde	Fruit	1670	1646	^86^
1-(2-furyl)-1,2-Butanediol	Sour cheese	1694	1563	^97^
3-methyl-2(5H)-Furanone	Cooked fat	1750	1713	^79^
2-Undecenal	Cheese	1780	1755	^98^
(E, E)-2,4-Decadienal	Fatty	1842	1819	^86^
Hexanoic acid	Barnyard, musty	1887	1849	^80^
2-methyl-1-Hexadecanol	Eggs	1900	**	**
Heptanoic acid	Sweaty	1991	1960	^84^
2-Pentadecanone	Stale	2046	2011	^83^
Octanoic Acid	Barnyard	2084	2070	^86^
Nonanoic acid	Stale, musty	2190	2169	^86^
n-Decanoic acid	Stale, musty	2280	2279	^80^

Compounds were tentatively identified using mass spectrometry and NIST database matching; compound confirmation was made using published retention index values. Tentative identification is indicated by “**”. Aroma descriptions were attributed via olfactory analysis (n = 3).

**Table 4:** GC/MS-O analysis of cultivated pork fat.

Compound	Aroma	RI (meas.)	RI (ref.)	References
2-ethyl-butanal	Butter	907	1018	^99^
3-methyl-3-buten-2-ol	Sweet, caramel	944	**	**
Pentanal	Sweet	980	979	^86^
Hexanal	Grass	1052	1078	^92^
2-pentylfuran	Sour	1177	1231	^89^
Octanal	Fatty, cooked fat	1251	1291	^86^
Dihydro-4,4-dimethyl-2(3H)-furanone	Fatty	1308	**	**
Nonanal	Fatty, grease	1353	1390	^89^
10-Methyl-8-tetradecen-1-ol acetate	Butter, popcorn	1392	**	**
(E)-2-Octenal	Nutty, cashews	1406	1434	^86^
1-Octen-3-ol	Musty	1456	1456	^86^
Acetic acid	Vinegar	1463	1465	^90^
10-Octadecenal	Pork fat	1466	1863	^100^
2-ethyl-1-Hexanol	Potato, starch	1491	1484	^101^
Benzaldehyde	Nuts	1537	1508	^78^
Dimethyl sulfoxide	Stale	1608	1582	^91^
d-Mannitol	stale, barnyard	1619	**	**
(Z)-2-Decenal	Cooked fat	1634	1627	^102^
Benzeneacetaldehyde	Fruit	1668	1646	^86^
Pentanoic acid	Cheese	1735	1744	^84^
9-Octadecen-12-ynoic acid, methyl ester	Cheese, sour	1746	**	**
2-methyl-hexanoic acid	Cheese	1749	1757	^103^
2-Undecenal	Cheese	1757	1755	^98^
Hexanoic acid	Barnyard, musty	1902	1849	^80^
Nonanoic acid	Stale, musty	2201	2169	^86^

Compounds were tentatively identified using mass spectrometry and NIST database matching; compound confirmation was made using published retention index values. Tentative identification is indicated by “**”. Aroma descriptions were attributed via olfactory analysis (n = 3).

## Discussion

The aroma profile of cell-cultivated adipose tissue was compared to that of conventional animal fat. The goal was to determine whether cultivated adipose can be considered as an additive to enhance the organoleptic properties of alternative meat products based on aroma. To achieve this, we established a porcine DFAT clonal cell line produced by single cell seeding. Cultivated adipocytes and conventional animal fat were then subjected to GC/MS and sensory evaluation studies to analyze the volatile chemistry profile and evaluate the consumer preference for each. Additionally, we conducted an analysis using GC/MS-O to identify compounds associated with specific positive and negative aroma attributes that were identified by sensory evaluation consumers.

The majority of stable preadipocyte cell lines are from rodents, with few preadipocyte cell lines established for other species^32^. To facilitate the study of pig fat for cellular agriculture, it is necessary to have access to an established adipogenic porcine cell line that can proliferate extensively and subsequently differentiate into mature adipocytes^33^. A variety of porcine adipocyte progenitor cells have been screened for adipogenesis such as stromal vascular cells (SVC), pluripotent stem cells, and adipose derived stem cells^25,34,35^. In our laboratory, the adipogenic potential of porcine SVCs has been compared to that of DFAT cells (data not shown). The DFAT cells were capable of accumulating more lipids during adipogenesis^25^. Therefore, porcine DFAT cells were used over SVCs for this project. Our previous work established a population of porcine DFAT cells which had a fatty acid composition closely resembling that of native pork fat. When grown in 2D, these cells could be aggregated into macroscale cultivated fat as a simplified method towards scalability. Using this method, we looked deeper into the utility of these cells for the formation of cultivated meat or more realistic meat alternatives.

We first established a porcine DFAT subclone from a previously isolated mixed population using single cell sorting and generated significant amounts of highly lipid laden adipocytes. Here, we show that among numerous successful single cell seedings, clone N47 demonstrated the highest intracellular lipid accumulation. However, N47 did not show more lipid accumulation than the original mixed population PDFAT. This was not expected, as previous studies showed that DFAT cells are often composed of more homogenous cell populations compared to stromal vascular and adipose derived stem cells due to their origin from highly homogenous fractions of mature adipocytes^36–38^. It is likely that although N47 was selected as the highest preforming clone among approximately 576 clones, this screening was not large enough to uncover a clone with significantly higher lipid accumulation capacity than the mixed population.

As reported in our previous study, the mixed population PDFAT lipid morphology is multilocular and N47 exhibits the same multilocular phenotype representing an immature white adipocyte^25^. Further culture optimization is required to achieve both higher lipid accumulation and unilocular lipids. In this study, optimization for PPARγ agonists dexamethasone, rosiglitazone and IBMX was conducted to improve lipid accumulation and promote a unilocular lipid phenotype. While limiting rosiglitazone to adipogenic induction media (first 2–3 days of adipogenesis) and lowering concentrations of IBMX to 0.1 M increased lipid accumulation capacity, intracellular lipids remained multilocular. We assessed the influence of ascorbic acid in adipogenic differentiation medium related to adipogenesis^39–43^. We found that ascorbic acid increased lipid accumulation in the cultivated porcine adipocytes. Additionally, we found that there was no significant difference in lipid accumulation when differentiating adipocytes in 10% and 1% serum. Thus, 10% FBS was used to differentiate adipocytes for GC/MS and sensory analysis to conserve yield, as serum has shown to increase cell adhesion. However, we did not find that the higher concentration of FBS prevented cell detachment throughout adipogenesis, therefore future studies may use 1% FBS to limit serum use^44^. Ultimately, further optimization to mimic extensive lipid accumulation as achieved in native porcine adipocytes should be explored further to maximize the system described in the present work.

After generating the adipocytes, the cells were mechanically aggregated via cell scraping to generate fat tissue and subsequently used for GC/MS analysis. Compounds of interest included volatile and semi-volatile compounds that would be released during the cooking process of pork products. Specifically, for molecules associated with fatty aromas, total ion chromatograms revealed that conventional porcine fat samples contained many long and medium-chain fatty acids such as oleic acid (9-octadecenoic acid), palmitic acid (n-hexadecanoic acid), capric acid (n-decanoic acid), myristic acid (tetradecanoic acid), and palmitoleic acid (hexadec-9-enoic acid). Our results largely agree with the literature, as numerous groups have reported palmitic acid and stearic acid as the most prominent saturated fatty acids and oleic acid as the most prominent unsaturated fatty acid in porcine fat^45–48^. Cultivated porcine fat samples contained mostly similar volatiles notably long chain fatty acids such as oleic acid, palmitic acid, and stearic acid. Other aroma attributes found in both samples such as “yeasty” and “cheesy” can be attributed to niacinamide (vitamin B3) and methyl ketones such as 2-Heptadecanone and delta-Dodecalactone, commonly detected in blue cheese^49–52^. It should be noted that dimethyl sulfoxide was detected in cultivated fat samples among the top 20 volatiles, likely because of using DMSO as a solvent to deliver small molecules such as dexamethasone and IBMX in adipogenic media. Further research comparing the aroma profiles of native fats with cultivated fats should seek alternative methods of incorporating hydrophobic compounds into the culture media to avoid any exogenous solvent additions like DMSO. For example, Intralipid uses lecithin as an emulsifier to deliver long-chain fatty acids and lecithin itself can be inductive of adipogenesis^24,53^.

Molecules associated with aroma attributes such as “rancid” were also detected in both profiles. Fat samples contained pentanal, 2-pentylfuran, octanal, and nonanal which correlate with oxidative sensory descriptors such as “rancid oil” in cooked meats^54,55^. Additionally, cultivated fat contained oxidation related volatiles that were not found in conventional fat, such as propanoic acid and 2-heptanone^56,57^. The addition of ascorbic acid and other antioxidants has shown to reduce lipid oxidation and sulfur-containing compounds in pork products^58,59^. Additionally, antioxidants may improve cell growth in serum-free conditions, which further emphasizes their addition when cultivating cells for food production^60^. Other methods such as gamma (γ) irradiation and electron beam irradiation have been investigated as methods for the selective removal of rancid and off-odors from food products. Both are non-invasive and food safe for human consumption, as they do not impact the favorable aromas, nutritional qualities, or overall appearance and acceptability of the product^61,62^. At high doses, γ irradiation may induce lipid oxidation and new off-odors, meaning further optimization of these methods is required to be applied to cultivated meat products^62^.

Sensory evaluation with human subjects is a key method for studying the unique characteristics of food products and is largely unexplored for cultivated meat^13^. Discrimination tasks are a simple sensory analyses which requires consumers to detect the difference between two samples. Here, we used discrimination testing to establish whether the aromas of cooked cultivated porcine fat could be distinguished from the aromas of cooked conventional porcine fat. The hypothesis tested was “with equivalent cooking parameters, are conventional and cultivated fat tissues perceptibly different products?”. Although the consumer panel was able to discriminate between the two samples, a significant preference for either fat sample was not found. A potentially confounding factor was the lack of extracellular matrix (ECM) and scaffolding for the in vitro-grown fat compared to native fat tissue. There is currently no information on the specific contribution of ECM to the aroma and flavor of meat products. Additionally, these relatively immature adipocytes utilized here may contribute to a different aroma profile compared to native porcine fat that consists of mature fat cells. Metabolic characterization of differentiating adipocytes with GC/MS methods indicates that metabolic changes induced during differentiation can be linked to the emission of different volatile and semi-volatile compounds^63,64^. Lipid metabolism also shifts as adipogenic differentiation progresses, resulting in more long-chain fatty acids and desaturation in later stages^63^. Therefore, aroma active volatiles at terminal stages of differentiation may have a different flavor profile than immature adipocytes. Additionally, in vitro-grown tissues were harvested and analyzed directly from culture whereas conventional meat samples underwent post-processing including carcass aging and long-term storage throughout the supply chain, which are known to significantly change the sensory characteristics of conventional meat^65^. Future work may evaluate in vitro-grown pork fat after mimicking such conditions or conventional pork fat at different stages of production.

CATA profiling was conducted to further understand the differences in the aroma character of conventional and cultivated fat from the consumer perspective^31^. Usage of attributes such as “fatty”, “savory”, “fried”, “meaty”, “bacon-like” and “roasted” were used equally by consumers to describe the two fat samples. Consumers used attributes “musty” and “barnyard” more often when describing cultivated porcine fat. As previously discussed, unlike cultivated porcine fat, commercial pork products undergo postmortem aging to increase the palatability attributes including aroma and flavor. This aging process leads to an increase in free amino acids and peptides which develops the “umami” flavor of pork via interactions with products of lipid oxidation such as peroxides. Additionally, products of lipid oxidation and nucleotide breakdown during this time are flavor precursors to the Maillard reaction, which contributes greatly to the browned, meaty aroma of cooked pork^66,67^.

GC/MS-O was used to evaluate the range of both pleasant and foul aromas in the two tissue samples. “Pleasant” odors refer to aromas that led to an average increase in overall liking and “foul” odors refer to aromas that led to an average decrease in overall liking as determined by the sensory consumers. Hexanoic acid, dimethyl sulfoxide, acetic acid and hexanal were detected in the top 20 volatiles from cultivated pork fat and appeared to also impact the sensory profile. Decanoic acid, 2-pentadecanone, hexanoic acid, nonanal, and acetic acid were detected in the top 20 volatiles from conventional pork fat and appeared to be sensory impactful. The sensory impact of DMSO in cultivated fat further supports the need to reduce the use of this agent in culture media and cryopreservation. With the exception of DMSO, these compounds have been previously reported in Yorkshire pork^68^.

Pleasant odors which occurred in both fat samples include fatty aldehydes such as pentanal, hexanal, octanal and nonanal. These compounds contributed to “sweet”, “buttery”, “fatty” and “greasy” aromas. Aldehydes are commonly known for their starchy, citrusy, fatty and waxy fragrance notes. In particular, C9 and C10 aldehydes such as nonanal and decanal detected in cultivated porcine fat are commonly found as skin odorants of various mammals resulting from the oxidation of sebaceous fatty acids^69^. These saturated aldehydes are typical products of lipid oxidation and induce an increase of rancid oil flavors in high concentrations^70^. However, these compounds presented a more positive, “fatty” aroma profile in the context of our pork fat samples. Foul odors detected in cultivated fat samples such as “barnyard”, “stale”, “musty” and “rancid oil” can be largely attributed to medium-chain fatty acid compounds such as pentanoic acid, hexanoic acid and heptanoic acid which are commonly created by the oxidation of the previously listed fatty aldehydes. These aldehydes along with furan compounds such as 2-pentylfuran and furanone found in both cultivated and conventional samples have been noted as the main components of pork flavor^71^. They may also impact meat flavor by interacting with components of the Maillard reaction.^72^.

With future efforts, investigating the impact of storage on the volatile compounds of in vitro-grown fat may be useful to determine if chilled aging results in removal of negative aromas and a more similar volatile profile to conventional pork. Another proposed solution to these odors, instead of removing them completely, would be to reduce their intensity until they express a more pleasant aroma. Volatiles such as pentane which is present in cultivated fat can be attributed to having a “barnyard” odor. Pentane, among other volatiles, can change its sensory description as the concentration of the chemical compound increases. At low levels, the compound is “beany” while at high levels it is considered “sweaty”, and at its highest levels it has a “barnyard” odor. Similarly, hexanal, which is present at a higher concentration in our cultivated porcine fat than native porcine fat, presents a “pea pod” odor at low levels while at high levels presents “rancid”, “sour” and “oxidized oil” aromatics^73–75^. Additionally, considering conventional pork fat contains many of the primary volatile markers for lipid oxidation due to either storage or cooking, reducing those in cultivated fat to equivalent levels should be sufficient. It’s important to note that even without these changes, consumers ultimately ranked cultivated and conventional porcine fat the same for overall liking.

## Conclusions

The primary objective of this paper was to address the following: What are the sensory characteristics of cultivated adipose tissue, and how do these compare to traditional animal-derived fat? Aroma profiles of cultivated adipose tissue were compared with conventional animal fat, aiming to evaluate in vitro-grown adipose as an additive to augment the organoleptic characteristics of alternative meat products. Through the establishment of a porcine DFAT clonal cell line and the application of advanced GC/MS techniques coupled with human sensory evaluations, we found that the volatile aroma compounds released during cooking were remarkably similar between the two fat sources. Despite some disparities in the presence and intensity of certain volatiles, the overall liking score from the sensory evaluation was similar for both in vitro and in vivo porcine adipose samples. The immature status of the adipocytes, the absence of extracellular matrix in the in vitro samples, the use of DMSO in the production process, and different post-harvest environments might be contributing factors to the differences in aroma profiles. Moving forward, our recommendations include refining the cell culture environment, investigating the role of extracellular matrix in aroma and flavor, and adjusting lipid supplementation during culture to better match the aroma profiles of conventional fat. As the field progresses, continued research is essential to not only closely replicate the aroma profile of native fats but also to ensure a complete and favorable sensory experience for consumers exploring alternative meats. Here we provided a methodological foundation to utilize and gain insight into these adipose sources. Importantly, the chemical-to-consumer links established by using this methodology can provide a guide to future studies on the topic. In addition, the data points to future opportunities to conduct molecular editing of flavors and odors to tailor outcomes, a major benefit to the processes involved in cultivated meat production.

## Methods

### Single cell seeding/cell line development

Porcine DFAT cells were isolated from the belly (subcutaneous fat) of a 93-day-old female Yorkshire pig (DOB: 10/18/2021)^25^. Single cell isolation from porcine DFAT cells was conducted at the Tufts University School of Medicine Flow Cytometry Core by fluorescence-activated cell sorting (FACS) with the FACSAria (BD Biosciences, Franklin Lakes, NJ, USA). Porcine DFAT cells were thawed at passage 3 and grown to 80% confluency in standard growth media containing Dulbecco’s Modified Eagle Medium (DMEM) + 20% Fetal Bovine Serum (FBS) + 100 μg/ml Primocin at 39 °C and 5% CO_2_. DFAT cells were detached using Accumax (AM105; Innovative Cell Technologies Inc, San Diego, CA, USA) and resuspended in standard growth media. Cells were strained using a 40 µm strainer and diluted to achieve a suspension concentration of 1 × 10^6^ cells/mL. Subsequently, approximately 576 single cells were isolated into six 96-well plates. Single cells were seeded into wells containing 3 different culture media formulations: 1) The “Normal” group contained growth media (DMEM + 20% FBS + 100 μg/ml Primocin). 2) The “Conditioned” group contained 50% growth media and 50% conditioned media, which was growth media that had been incubated with DFAT cells for 2–3 days. 3) The “Vitronectin” group contained growth media supplemented with vitronectin peptide (VTN-N, 0.25 μg/cm^2^). Three days after seeding, wells were visually screened for the presence of single cells, or a single colony/cluster of several cells using an inverted light microscope (Olympus CKX53; Evident Life Science, Shinjuku-ku Tokyo, Japan). After eight days, cells were screened and VTN-N supplemented colonies were omitted from further screening due to poor proliferation. Wells in normal and conditioned media with successfully sorted cells were dissociated from the 96-well plates once a dense colony/cluster formed, to prevent the development of localized areas of cell confluence. After this initial passage, cells were cultured to 70–80% confluency and progressively moved to 24-well, 12-well, and 6-well plates. Cells were regularly inspected with a microscope for cell density and fat accumulation throughout six days of adipogenesis. Intracellular lipid accumulation of all viable clones was quantified with Oil Red O Stain Kit (ab150678; Abcam, Cambridge, UK) on day six (Supplementary Fig. S1). Five clones N18, N20, N47, C15, and C34 were selected based on previous proliferation screening and lipid accumulation screen (data not shown). Cells were monitored for nine passages before reducing the screen to clones N18, N47, and C34 based on lipid accumulation (data not shown). Clone N47 was finally selected based on a comparison of lipid accumulation for these top three clones as well as the mixed population PDFAT (Supplementary Fig. S2).

### Preadipocyte cell culture and adipogenic differentiation

Long term cell culture continued with PDFAT clone N47 grown using DMEM + 20% FBS + 100 µg/ml Primocin + 0.25 μg/cm^2^ laminin 511-E8 (N-892021; Iwai North America Inc., San Carlos, California, USA). Laminin 511–58 was used for ECM supplementation at every passage. The cells were cryopreserved in 90% culture medium and 10% dimethyl sulfoxide (DMSO). Cells were cultured in standard plasma treated tissue culture flasks and were handled gently during cell feedings to prevent detachment of adipocytes. During the aspiration of spent culture media, culture flasks were inverted and media was collected from the roof and sidewalls of the flasks. Additionally, new media was dispensed in the inverted position to avoid pipetting directly on the cells. The flasks were slowly returned to their upright positions to ensure gentle exposure of new media to the cells.

Porcine preadipocytes were grown until 100% confluency in culture medium. After remaining confluent for at least 24 h, cells were switched to adipogenic induction media consisting of Advanced DMEM/F12 (12,634–028; Thermo Fisher) with 10% FBS, 100 µg/ml Primocin, 500 µg/ml Intralipid (I141; Sigma), 0.1 mM 3-isobutyl-1-methylxanthine (IBMX, I5879; Sigma), 5 μM rosiglitazone (ROG, R0106; Thermo Fischer), 0.5 μM dexamethasone (DEX, AC23030; Thermo Fisher), 2 mM (1 ×) GlutaMAX (35050-061; Thermo Fisher), 20 µM biotin (B04631G; TCI America, Portland, OR, USA), and 10 µM calcium-D-pantothenate (P001225G; TCI America). Cells remained in adipogenic induction media for 2 days. During lipid accumulation, the same medium was used, omitting IBMX and ROG. Cells remained in the lipid accumulation media for 9–11 days. Media optimization was conducted for 5 μM rosiglitazone, 0.5 μM dexamethasone, 0.1 mM IBMX, 20 ng/mL bone morphogenic protein-4 (BMP4, NBC119056; Thermo Fisher), and 113 µM ascorbic acid (57-785-0; Fischer Scientific).

### Fat harvest

After adipogenesis, the spent culture media was aspirated by gentle inversion of the flask as previously described. Adipocytes were rinsed with Dulbecco’s phosphate buffered saline (DPBS) 3–5 times and flasks were kept vertical for 1–2 min to thoroughly drain DPBS and any remaining media. Once excess DPBS was aspirated, the adipocytes were harvested using a cell scraper (83.3952; Sarstedt, Nümbrecht, Germany) via raking motions from the front to the back of the flask. Periodically, additional liquid was gently aspirated from the flask without removing the scraped cells. The cell mass was pushed to the back of the flask and collected, then transferred into a pre-weighed 0.6 ml Eppendorf tube. Samples were stored at − 80 °C.

### Lipid staining

Cultured adipocytes in 96-well tissue culture well plates were stained to confirm intracellular lipid accumulation. Here, PDFAT N47 adipocytes were stained using Oil Red O Stain Kit (ab150678; Abcam, Cambridge, UK). Cells were rinsed twice with DPBS and fixed with 4% paraformaldehyde (PFA) for 20 min at room temperature. Oil Red O assessment was conducted according to the manufacturer’s protocol. Media and other liquids were aspirated with a pipette to avoid detachment of adipocytes. Cells were incubated with propylene glycol at RT for 5 min and Oil Red O was heated to 60 °C. Propylene glycol was replaced by warmed Oil Red O and incubated at RT for 7 min. Cells were incubated with 85% propylene glycol in distilled water for 1 min and rinsed twice with distilled water. The results for Oil Red O staining were analyzed on a microplate reader at 515 nm.

### Native porcine adipose samples

Pork fat tissue (*Sus scrofa domesticus*) from the belly was purchased commercially from a wholesale distributor. Samples were displayed at refrigerator temperatures in the store and transported to the laboratory at RT. The tissue was sliced, preserved with a food grade vacuum sealer and stored at − 20 °C. Subcutaneous fat from tissue samples was cut at room temperature and used for GC/MS, GC/MS-O, and sensory evaluation experiments.

### GC/MS

Cultivated fat samples were thawed from − 80 °C and weighed at RT. Conventional pork belly was thawed from − 20 °C and subcutaneous fat was sliced from the sample. Next, 50 mg samples of each tissue were weighed into a 20 mL round bottom amber glass vials (Restek, Bellefonte, PA).

The volatile profile of each tissue was collected using an Agilent 7890A gas chromatograph coupled with an Agilent 5977A mass selective detector (Agilent Technology, Santa Clara, CA, USA). The GC/MS was equipped with a robotic multi-purpose autosampler (MPS), dynamic headspace unit (DHS) thermal desorption unit (TDU), and cooled injection system (CIS4), each manufactured by Gerstel (Gerstel, Linthicum, MD). The sampling protocol began with the MPS transferring the 20 mL vial into the incubation chamber of the DHS unit. The tube was first incubated at 120 °C for 15 min; the vial was then purged with 200 mL of nitrogen at a flow of 100 ml/min; volatiles were trapped onto a Tenax filled TDU tube with 1,500 mL of nitrogen at a flow rate 100 mL/min at 120 °C; the Tenax trap was then dried with 200 mL of nitrogen at 50 mL/min; lastly, the Tenax filled TDU tube was then transferred to the TDU for desorption. Prior to desorption, the CIS with glass bead liner, was cooled to − 120 °C with liquid nitrogen. Desorption was initiated by ramping the TDU from 40 °C to 300 °C at 720 °C/min with a 5 min hold at 300 °C. After desorption the TDU tube was removed from the TDU. The chromatographic analysis was initiated with the ramping of the CIS from − 120 °C to 280 °C followed by a 5 min hold. The 7890A was outfitted with a DB-WAX UI capillary column (30 m, 0.25 mm i.d., 0.25 mm film thickness (Agilent Technologies, Santa Clara)). The 7890A oven temperature was programmed to hold at 40 °C for 1 min; then ramp at 10 °C/min to 250 °C, and then hold 250 °C for 10 min. The helium carrier gas was set to a constant flow 1.2 mL/min. The MSD was set to a solvent delay for the initial 1.25 min, with an electron energy of − 70 eV, a source temperature of 250ºC, and quadrupole temperature of 150ºC. Data was acquired in scan mode ranging from 35 m*/z* to 300 m*/z*. Triplicate injections of each sample were completed and unknown compounds were identified via matching MS fragmentation patterns against the NIST17 mass spectral libraries and published retention index values.

### GC/MS-O

Preparation of tissue samples for the GC/MS-O are identical as described for the GC/MS (see GC/MS). Olfactory data was collected by Emily T. Lew. The assessor remained at the sniffing port for the duration of the sample run, approximately 45 min. To avoid sensory fatigue, the analysis was conducted in an ambient environment free of strong odors and the assessor was separated from the surrounding environment. Samples were analyzed with 30 min intervals and no more than three samples per day were analyzed.

Olfactory data was collected using an Agilent 7890A coupled with an Agilent 5975C mass selective detector (Agilent Technology, Santa Clara, CA, USA). The GC/MS was equipped with an MPS Robotic autosampler, DHS TDU, cooled injection system (CIS4), and olfactometer detection port (ODP3), each manufactured by Gerstel (Gerstel, Linthicum, MD). Tissue samples were processed using an equivalent DHS, TDU, and CIS settings as described for the GC/MS (see GC/MS). Chromatographic separation was achieved using an Agilent INNOWAX capillary column (30 m, 0.25 mm i.d., 0.25 mm film thickness (Agilent Technologies, Santa Clara)), with an equivalent oven temperature program.

### Sensory panel recruitment

A total of 55 consumers were recruited from the students and staff in the Tufts School of Engineering, Medford, Massachusetts. Consumers were recruited via flier advertisements and email. Email databases included all undergraduate and graduate students in the Tufts School of Engineering. All consumers received information regarding test type, product to be tested, and any risks or benefits of participating in the study. To participate, consumers had to be 18 years or older and willing to smell in vitro and conventional porcine fat tissues. Prior to each test, consumers were provided with consent forms and given another brief description of the samples and testing method. The study assumed consumers were strictly untrained. Consumers who were considered untrained had no experience participating in either a triangle or check-all-that-apply sensory evaluation study. The entire study contained approximately 47% male, 47% female, and 6% non-binary identifying consumers.

### Triangle test

To determine if subjects could discriminate between cultivated and conventional porcine fat samples, a triangle test was conducted^76,77^. In vitro and conventional porcine fat samples were thawed from frozen at − 80 °C and − 20 °C samples, respectively. One-hundred mg samples were placed in headspace vials with the caps on and heated to 120 °C in a commercial oven for 45 min. Sample vials were warmed in 60 °C bead baths throughout the duration of the sensory evaluation. This study adhered to guidelines outlined by the American Society for Testing and Materials (ASTM) in their “Standard Guide for Serving Protocol for Sensory Evaluation of Foods and Beverages” (E1871-17) which recommends a minimum holding temperature of 57 °C for the prevention of microbial growth. Recommended pretesting was conducted where sample aroma at a holding temperature of 60 °C was evaluated for up to 6 h. It was determined that no noticeable change in odor or rancid off-odors developed during this time that would prevent an untrained consumer from completing the discrimination task.

Data was collected in one day over a 6-h period, during which five sessions with a maximum of twelve consumers per session were tasked with differentiating between cultivated and conventional porcine fat aroma. A total of 55 consumers completed the evaluation. Consumers completed the session at an isolated desk with natural lighting. Each consumer was asked to assess one triangle set containing three fat samples. Samples of the adipose tissues were presented at random, making combinations such as AAB, ABA, BAA, BBA, BAB, and ABB. All six combinations of conventional and invitro porcine fat samples were presented throughout the study. Vials containing samples were concealed to mask visual differences between fat tissues. Each triangle was presented in a series along with a cup of water.

### Consumer preference and CATA

Sample preparation for the check-all-that-apply (CATA) analysis was identical to that of the triangle test. Separate samples were used for the triangle test and CATA analysis.

The 55 consumers provided preference information through a questionnaire which used a 7-point hedonic scale and CATA task.

Two fat samples were presented in random order for every consumer. The ballot questions were as follows:

- Two questions using the 7-point hedonic scale ranging from Extremely Dislike to Extremely Like: “Please indicate your liking of the aroma found in Vial X.”
- Two check-all-that-apply (CATA) questions: “Which attributes would you use to describe the aroma found in Vial X? Check all that apply” from a list of the following descriptors: Meaty, Fresh Meat, Bacon-like, Fatty, Savory, Cheesy, Sweet, Fruity, Roasted, Rancid, Fried, Animal, Barnyard, Sweaty, Metallic, Cardboard, Sour, Musty.
- After samples were evaluated, consumers completed an exit survey for the following variables: “Age,” “Gender”, divided into four possible categories; “Income”, divided into nine possible categories; “Education”, six categories; “Would you be willing to buy cultivated meat if it has the same price as conventional meat?”, Yes or No; Would you be willing to pay 10% more?”, Yes or No; Would you be willing to buy it with a 10% discount?”, Yes or No; “What do you like about the idea of cultivated meat?”, “What do you dislike about the idea of cultivated meat?”. A complete summary of consumer exit survey responses can be found as Supplementary Table S6.

### Statistical analysis

Statistical analyses were conducted with GraphPad Prism 10.1.1 and R version 4.3.2. The specific analyses include Analysis of variance (ANOVA) with Tukey’s post-hoc tests and Fisher's exact tests. Error bars and ± ranges represent standard deviations. A p-value of 0.05 was used for statistical significance. All experiments in this study were carried out with at least triplicate samples (n ≥ 3). Principal component analysis was applied to the scaled volaille data using the FactoMine R package with 95% confidence ellipses drawn using the *coord.ellipse* function.

### Ethics declaration

Isolation of adipocyte progenitor cells from a 93-day-old female Yorkshire pig (*Sus domesticus)* was approved by the Tufts University Medical Center and sourced from the Tufts Comparative Medicine Services (CMS). CMS methods are in accordance with the guidelines of the United States Department of Agriculture (USDA), Office of Laboratory Animal Welfare (OLAW), Massachusetts Department of Public Health (MDPH) and Association for Assessment and Accreditation of Laboratory Animal Care International (AAALAC). Additionally, all methods were conducted in accordance with ARRIVE guidelines 2.0.

The sensory evaluation study was approved by the Tufts University Health Sciences Institutional Review Board (IRB) for research involving human subjects (Study 00003031). All experiments were performed in accordance with the Tufts University Health Sciences IRB guidelines for exempt studies. Informed consent was obtained from all consumers.

### Supplementary Information

Supplementary Information.

## Data Availability

The GC/MS datasets generated during and/or analyzed during the current study are available from the corresponding author on request.
