# Supplementary material for: Chemical and sensory analyses of cultivated pork fat tissue as a flavor enhancer for meat alternatives
Source: Sci Rep. 2024 Jul 31;14:17643. doi: 10.1038/s41598-024-68247-4 (PMC11291926; doi:10.1038/s41598-024-68247-4)
Supplement: Supplementary file 1 — Supplementary Information. [file 41598_2024_68247_MOESM1_ESM.pdf]

## Supplementary Information

# Chemical and Sensory Analyses of Cultivated Pork Fat Tissue as a Flavor Enhancer for Meat Alternatives

Emily T. Lew[1], John S.K. Yuen Jr.[1], Kevin L. Zhang[2], Katherine Fuller[3], Scott C. Frost[2], David L. Kaplan[1*]

[1]Tufts University School of Engineering, Medford, MA 02155, USA

[2]Tufts University School of Arts and Sciences, Medford, MA 02155, USA

[3]Tufts University Friedman School of Nutrition, Boston, MA 02111, USA

*Corresponding author: david.kaplan@tufts.edu

## Supplementary Figure S1: Clonal isolation adipogenesis screening

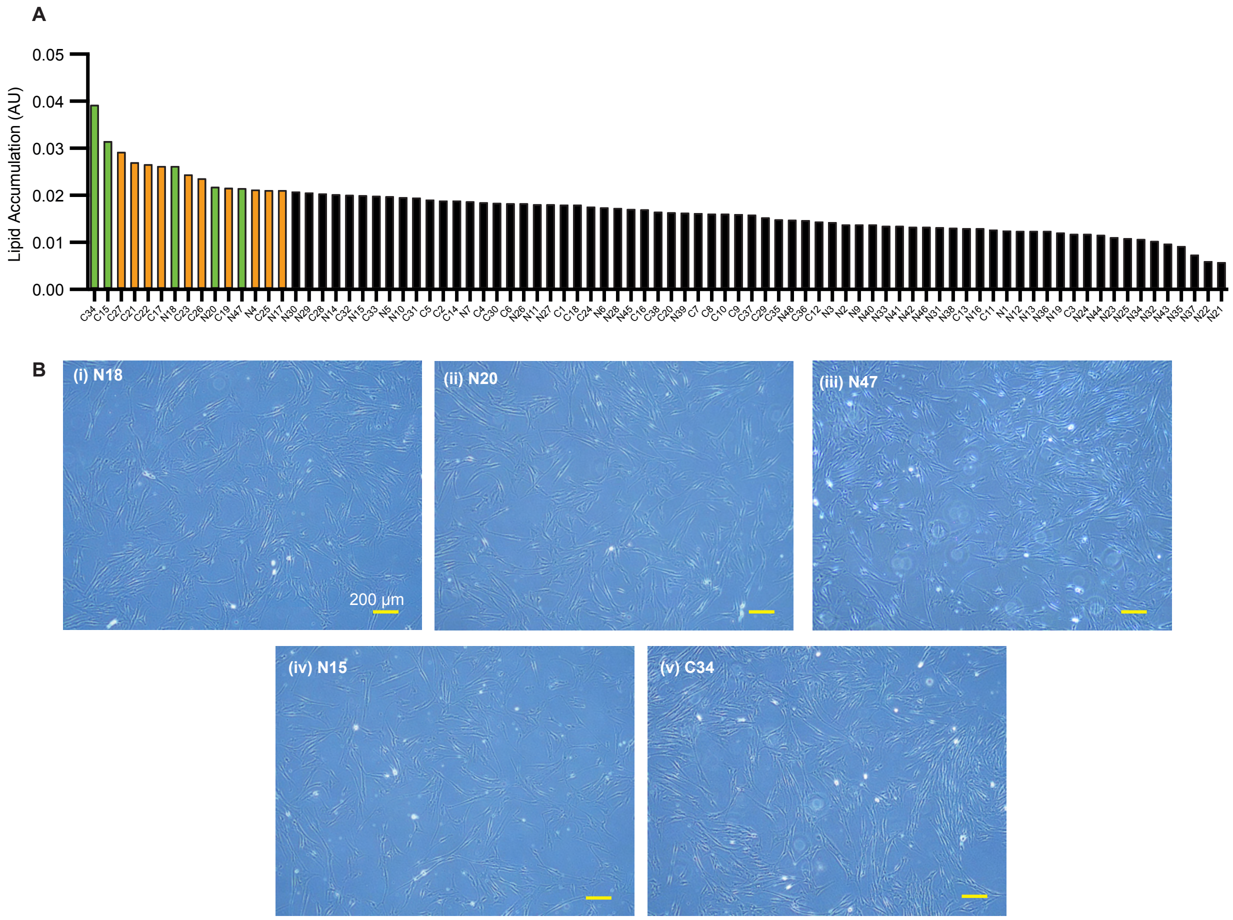


**Supplementary Figure S1.** Screening of clones isolated from mixed population PDFAT. (**A**) Lipid accumulation screen of normal and conditioned media clones. Clones N1-N48 and C1-C38 were stained with Oil Red O. All clones in vitronectin supplemented media were omitted due to poor proliferation. Clone N8 was omitted due to absorbance value 0. Top 15 clones are highlighted (orange) and clones picked for further screening are highlighted (green). Five clones were selected based on lipid accumulation and previous proliferation screening. (**B**) Morphology of top five clonal populations during proliferation (**i**) N18 (**ii**) N20 (**iii**) N47 (**iv**) C15 (**v**) C34 (passage 3). Scale bar represents 200 µm for all images. Scale bar represents 200 µm for all images.

**Supplementary Figure S2: Screening of clones N18, N47 and C34**

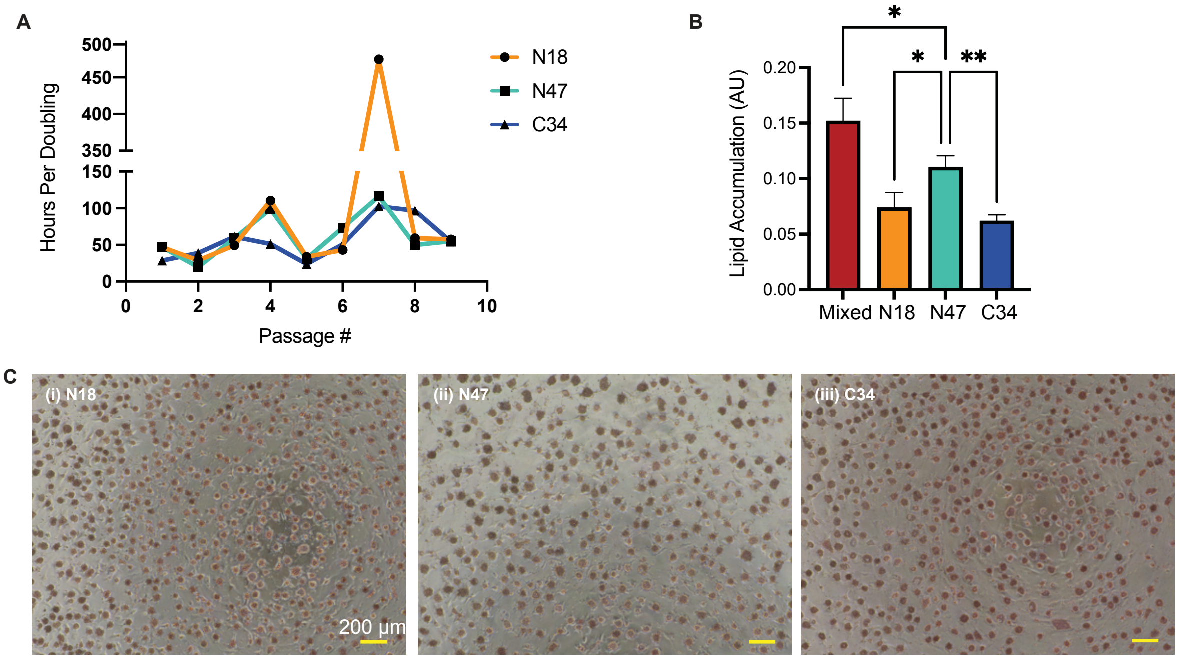


**Supplementary Figure S2.** Screening of clones N18, N47 and C34. (**A**) Hours per doubling (doubling time) of top three clones N18, N47 and C34 up to passage 9 (**B**) Lipid accumulation screen of mixed population PDFAT and top three clones (P8) determined by proliferation speed up to P6. Screening continued with clone N47. (**C**) Clones (**i**) N18 (**ii**) N47 (**iii**) C34 after six days of adipogenesis and stained with Oil Red O. Scale bar represents 200 μm for all images.

**Supplementary Figure S3: PDFAT lipid morphology**

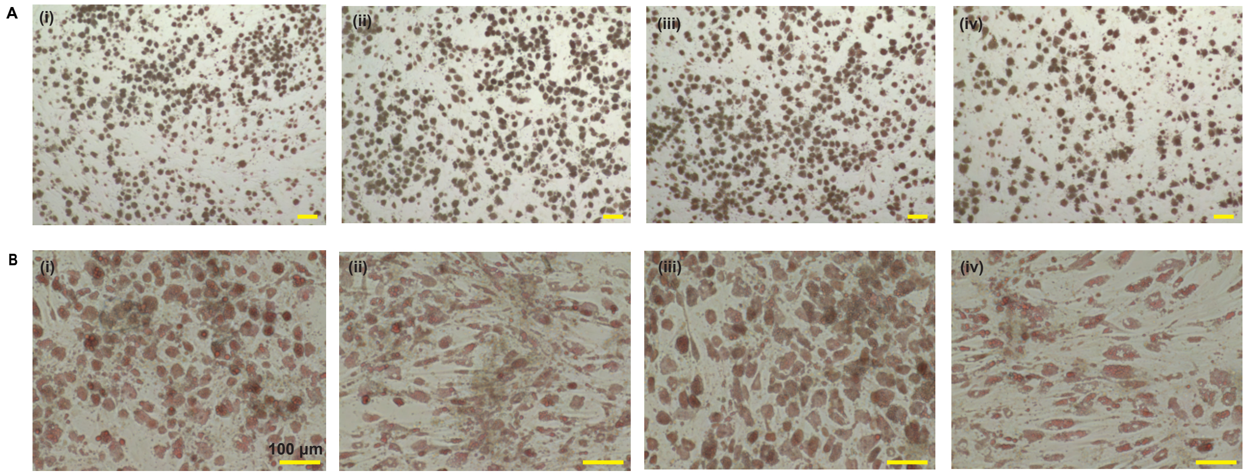


**Supplementary Figure S3.** Lipid morphology of N47 clones and PDFAT mixed population throughout IBMX, dexamethasone and rosiglitazone media optimization efforts. (**A)** Morphology of N47 cells with various concentrations of IBMX present in the induction media (P12) (A,**i**) 0 mM (A,**ii**) 0.1 mM (A,ii**i**) 0.25 mM (A,**iv**) 0.5 mM. Scale bar represents 100 µm (**B**) Morphology of mixed population PDFAT (B,**i**) +DEX +ROG (**B,ii**) –DEX +ROG (**B,iii**) +DEX –ROG (**B,iv**) –DEX –ROG in accumulation media (P3). Scale bar represents 100 µm.

**Supplementary Figure S4: N47 media optimization**

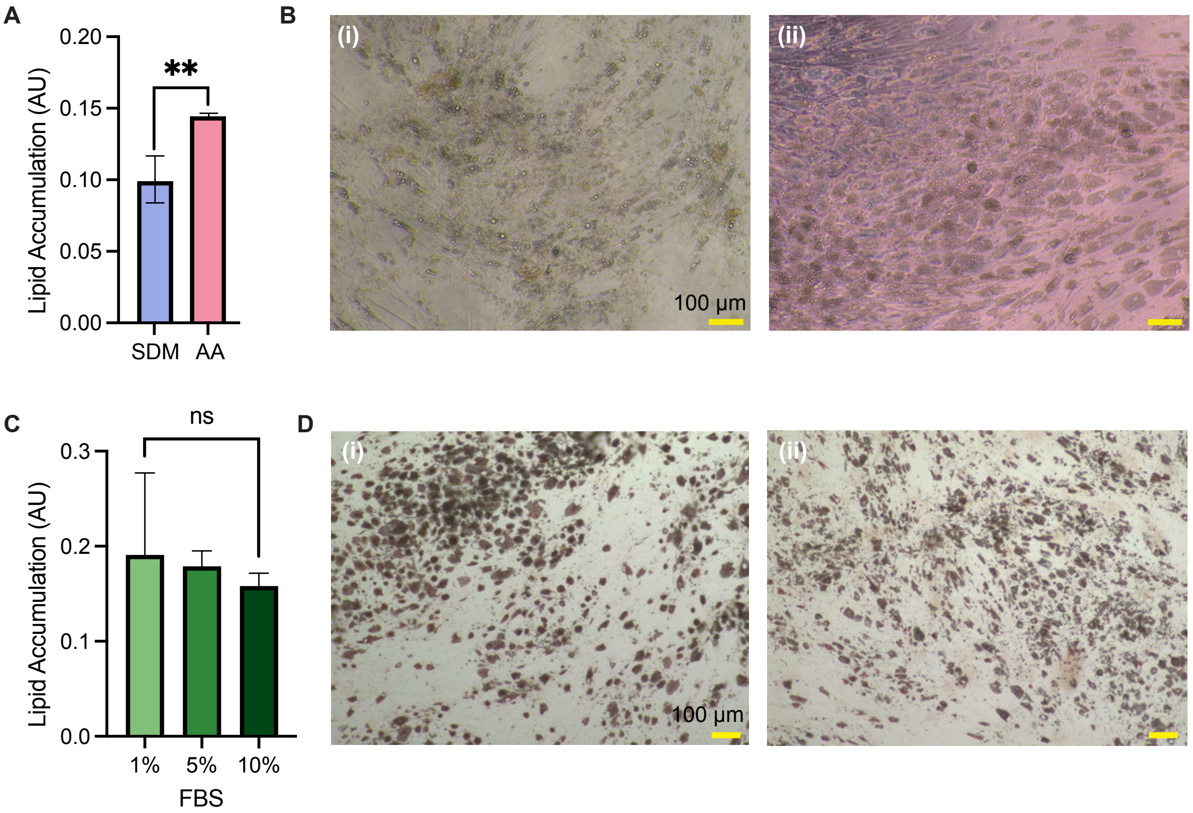


**Supplementary Figure S4.** Ascorbic acid adipogenic media screen for clone N47. (**A**) Lipid accumulation screen where SDM= standard growth media and AA = SDM supplemented with 113 µM ascorbic acid as described in Jurek et al. (n= 3). (**B**) N47 (passage 9) after nine days of adipogenesis with (**i**) SDM and (**ii**) AA. Scale bar represents 100 µm (n=4). **(C)** FBS concentration in induction and lipid accumulation medias with clone N47 (passage 23). (**D**) N47 (passage 23) after 10 days of adipogenesis with (**i**) 1% FBS and (**ii**) 10% FBS. Scale bar represents 100 µm (n=3).

**Supplementary Table S1: Total compound comparison of conventional and cultivated porcine fat.**

(**) tentatively identified with NIST hit.

| Compound | Peak Area | | Retention Time (mins.) | | RI (meas.) | |
|---|---|---|---|---|---|---|
| | In vitro | In vivo | In vitro | In vivo | In vitro | In vivo |
| 1-Hexadecanol | 8850120 | 3221414 | 19.3 | 20.4 | 2369 | 2503 |
| 1-Pentanol | 3871048 | 3163793 | 20.1 | 6.9 | 2464 | 1258 |
| 2-Heptadecanone | 2419961 | 9581884 | 18.1 | 18.1 | 2236 | 2237 |
| 2-Octenal, (E)- | 3073749 | 3885336 | 8.8 | 9.3 | 1392 | 1434 |
| 2,2,4-Trimethyl-1,3-pentanediol diisobutyrate | 5508359 | 15084606 | 6.6 | 14.7 | 1234 | 1887 |
| 2H-Pyran-2-one, tetrahydro-6-nonyl- | 2419013 | 3431779 | 15.8 | 21.8 | 1997 | 2672 |
| 2H-Pyran-2-one, tetrahydro-6-undecyl- | 7349251 | 7576589 | 12.9 | 23.8 | 1721 | 2901 |
| 3-Nonen-2-one | 2457239 | 2917201 | 14.6 | 16.7 | 1877 | 2082 |
| 9-Octadecenoic acid, (E)- | 32767970 | 142004120 | 22.4 | 27.1 | 2741 | 3066 |
| Acetic acid | 27279476 | 9404231 | 10.4 | 10.4 | 1517 | 1518 |
| Acetic acid, methyl ester | 2326433 | 5073681 | 13.3 | 7.6 | 1763 | 1303 |
| Benzaldehyde | 1260470 | 3028198 | 12.1 | 10.5 | 1655 | 1526 |
| Butanoic acid | 3649199 | 5888981 | 11.8 | 11.8 | 1626 | 1626 |
| Butyrolactone | 4176999 | 2563600 | 11.8 | 11.8 | 1626 | 1633 |
| delta-Dodecalactone | 3981136 | 7688113 | 11.9 | 19.9 | 1643 | 2439 |
| Dibutyl phthalate | 7575945 | 3231474 | 15.6 | 22.0 | 1972 | 2703 |
| Dimethyl ether | 1757173 | 3870217 | 14.0 | 3.1 | 1820 | 863 |
| Furan, 2-pentyl- | 27107789 | 4858058 | 6.6 | 6.6 | 1234 | 1233 |
| Hexadecane | 8383065 | 2130597 | 11.5 | 11.4 | 1600 | 1599 |
| Hexanal | 18069051 | 6835885 | 4.7 | 4.6 | 1093 | 1089 |
| Hexanoic acid | 37043963 | 16083840 | 14.2 | 14.2 | 1844 | 1844 |
| n-Hexadecanoic acid | 57742859 | 145163036 | 23.9 | 23.9 | 2907 | 2908 |
| Niacinamide | 27397941 | 19010696 | 24.6 | 24.6 | 2972 | 2971 |
| Nonanal | 5291328 | 11334456 | 8.9 | 8.8 | 1396 | 1395 |
| Nonanoic acid | 7724917 | 9881773 | 17.5 | 17.5 | 2165 | 2165 |
| Octadecanoic acid | 20376824 | 48567818 | 26.6 | 26.6 | 3092 | 3092 |
| Octadecanoic acid, ethyl ester | 2988928 | 1915540 | 20.1 | 20.1 | 2464 | 2464 |
| Octanal | 4156389 | 5552584 | 7.4 | 7.4 | 1291 | 1291 |
| Octanoic acid | 6628059 | 45795310 | 16.4 | 16.4 | 2059 | 2058 |
| Palmitoleic acid | 8674152 | 73204134 | 24.3 | 24.3 | 2943 | 2944 |
| Pentadecanoic acid | 2683743 | 3395664 | 22.9 | 22.9 | 2800 | 2801 |
| Pentanal | 6715416 | 3474073 | 3.5 | 3.5 | 886 | 887 |
| Phthalic acid, isobutyl 4-octyl ester | 8077736 | 3601691 | 208.0 | 20.8 | 2548 | 2547 |
| Tetradecane | 16025271 | 1949950 | 8.9 | 8.9 | 1400 | 1399 |
| Tetradecanoic acid | 13778200 | 86431341 | 22.0 | 22.0 | 2695 | 2696 |

**Supplementary Table S2. Compounds unique to cultivated porcine fat samples**
(**) tentatively identified with NIST hit.

| Compound | Peak Area | Retention Time (mins.) | RI (meas.) |
|---|---|---|---|
| (E)-9-Octadecenoic acid ethyl ester | 7758554 | 19.9 | 2439 |
| (Z)-9-octadecen-4-olide | 3470941 | 27.1 | 3066 |
| 1-Butanamine, 2-methyl-N-(2-methylbutylidene)- | 2132580 | 20.3 | 2485 |
| 1-Butanamine, N-butylidene- | 4052708 | 26.4 | 3068 |
| 1-Butene, 3-butoxy-2-methyl- | 3501644 | 5.4 | 1147 |
| 1-Dodecanol | 3944123 | 6.2 | 1203 |
| 1-Tetradecanol | 2733637 | 9.6 | 1452 |
| 1H-Benzocycloheptene, 4,4a,5,6,7,8,9,9a-octahydro-4a-methyl-, trans- | 3304041 | 6.9 | 1257 |
| 2-Decanone | 22348937 | 17.6 | 2178 |
| 2-Fluoroaniline, N-pentyl- | 3825081 | 10.2 | 1497 |
| 2-Heptanone | 32461734 | 19.5 | 2394 |
| 2-Nonanone | 21354871 | 6.0 | 1189 |
| 2-Pentanol, 2,4-dimethyl- | 2425799 | 9.3 | 1434 |
| 2,6-Di-tert-butyl-4-hydroxy-4-methylcyclohexa-2,5-dien-1-one | 3480077 | 14.7 | 1887 |
| 2(3H)-Furanone, 5-butyldihydro- | 2372591 | 15.8 | 1997 |
| 2(3H)-Furanone, dihydro-3-hydroxy-4,4-dimethyl-, (.+/-.)- | 4671823 | 16.8 | 2099 |
| 3-Octen-2-one | 3036754 | 23.8 | 2901 |
| 4-Oxohex-2-enal | 3453521 | 14.6 | 1877 |
| 4H-Pyran-4-one, 2,3-dihydro-3,5-dihydroxy-6-methyl- | 8190230 | 9.1 | 1411 |
| 6-Undecanol | 3480872 | 23.7 | 2887 |
| 7,9-Di-tert-butyl-1-oxaspiro(4,5)deca-6,9-diene-2,8-dione | 2686252 | 13.3 | 1764 |
| Acetamide | 2594161 | 13.3 | 1763 |
| Acetoin | 9292528 | 10.4 | 1517 |
| Acetophenone | 1414578 | 7.6 | 1302 |
| Amantadine | 2507091 | 7.4 | 1290 |
| Benzene, 1,1'-(1,2-cyclobutanediyl)bis-, cis- | 3222745 | 11.7 | 1621 |
| Benzoic acid | 5015234 | 19.7 | 2412 |
| Cyclodecane | 3736136 | 11.8 | 1633 |
| Cyclohexanone, 2-butyl- | 2507879 | 14.0 | 1820 |
| Dimethyl Sulfoxide | 31348131 | 3.1 | 864 |
| Ethanone, 1-(3-butyloxiranyl)- | 10646196 | 11.9 | 1643 |
| Heptadecane | 3206681 | 12.6 | 1699 |
| Hexadecanoic acid, ethyl ester | 3842177 | 18.3 | 2258 |

| | | | |
|---|---|---|---|
| Methane, isocyanato- | 10031276 | 2.9 | 850 |
| n-Pentadecanol | 7450026 | 18.5 | 2280 |
| Pentanoic acid | 4933703 | 13.0 | 1737 |
| Pentanoic acid, 5-hydroxy-, 2,4-di-t-butylphenyl esters | 3068763 | 18.8 | 2306 |
| Phthalic acid, di(2-propylpentyl) ester | 15974451 | 27.5 | 3175 |
| Propanal, 2-methyl- | 11318251 | 2.3 | 804 |
| Propanoic acid | 10145153 | 10.7 | 1537 |

**Supplementary Table S3. Compounds unique to conventional pork fat**
(**) tentatively identified with NIST hit.

| Compound | Peak Area | Retention Time (mins.) | RI (meas.) |
|---|---|---|---|
| 1-Octen-3-ol | 3399388 | 9.6 | 1452 |
| 2-Decenal, (E)- | 5990927 | 12.0 | 1648 |
| 2-Heptenal, (E)- | 6872204 | 7.9 | 1328 |
| 2-Nonenal, (E)- | 4614505 | 10.7 | 1540 |
| 2-Pentadecanone | 54228020 | 16.1 | 2027 |
| 2-Tridecanone | 6861489 | 13.9 | 1813 |
| 2-Undecenal | 5719360 | 13.3 | 1757 |
| 2,4-Decadienal | 8272301 | 13.9 | 1815 |
| 2,4-Imidazolidinedione, 1-methyl- | 2932071 | 22.2 | 2718 |
| 2(3H)-Furanone, 5-acetyldihydro- | 3805962 | 16.5 | 2063 |
| 2(5H)-Furanone, 3-methyl- | 3999106 | 12.9 | 1721 |
| 2H-Pyran-2-one, tetrahydro-6-pentyl- | 8581889 | 17.8 | 2205 |
| 3-Methyl-hexanoic acid | 3195677 | 15.4 | 1951 |
| 3(2H)-Furanone, 4-hydroxy-5-methyl- | 3154759 | 17.0 | 2118 |
| 9-Decenoic acid | 28614654 | 19.0 | 2332 |
| 9(E),11(E)-Conjugated linoleic acid | 59967809 | 28.1 | 32345 |
| Benzeneacetaldehyde | 6169171 | 12.0 | 1643 |
| Butanoic acid, 3-methyl- | 4842018 | 12.3 | 1669 |
| cis-10-Heptadecenoic acid | 5391139 | 25.6 | 2992 |
| Dimethyl sulfone | 2797316 | 9.0 | 1409 |
| Dodecanoic acid | 42205675 | 20.3 | 2483 |
| Ethanol, 2-(2-butoxyethoxy)- | 2586821 | 13.8 | 1800 |
| Ethanol, 2-(hexyloxy)- | 3040865 | 11.6 | 1616 |
| Formic acid, octyl ester | 2336234 | 11.0 | 1562 |
| Glycerin | 51668188 | 18.9 | 2316 |
| Heptanal | 4260583 | 6.0 | 1189 |
| Myristoleic acid | 5472385 | 22.4 | 2741 |
| n-Decanoic acid | 111782473 | 18.5 | 2272 |
| Oxacyclotridecan-2-one | 3389781 | 20.8 | 2544 |
| p-Cresol | 3327913 | 16.6 | 2079 |
| Pentadecane | 2704867 | 10.2 | 1499 |
| Phenol, 4-(1-methylpropyl)- | 2591936 | 14.3 | 1852 |
| Propanoic acid, 2-methyl-, 3-hydroxy-2,2,4-trimethylpentyl ester | 9972895 | 14.5 | 1873 |
| Thiophene, 2,3-dihydro- | 5896415 | 19.4 | 2384 |

**Supplementary Table S4: Triangle test with 54 consumers.**

| Unalike Triangle Sample | Number of Correct Responses |
|---|---|
| Pork Belly Fat | 20/24 |
| Cultured Pork Fat | 21/30 |
| Total | 41/54[α] |

[α] Critical number of correct responses required to demonstrate statistical significance ($p<0.05$) in a triangle test is 25/54 [40].

**Supplementary Table S5: Full triangle test summary**

Consumers were presented with one triangle set consisting of 3 samples. Response of “1” indicates successful discrimination of the unalike sample while “0” indicates unsuccessful discrimination. N/A indicates no answer was given.

| Consumer | Discrimination |
|---|---|
| 1 | 1 |
| 2 | 0 |
| 3 | 1 |
| 4 | 1 |
| 5 | 1 |
| 6 | 1 |
| 7 | 0 |
| 8 | 1 |
| 9 | 1 |
| 10 | 1 |
| 11 | 1 |
| 12 | 1 |
| 13 | 0 |
| 14 | 1 |
| 15 | 1 |
| 16 | 1 |
| 17 | 1 |
| 18 | 1 |
| 19 | 1 |
| 20 | 1 |
| 21 | 1 |
| 22 | 0 |
| 23 | 0 |
| 24 | 1 |
| 25 | 0 |
| 26 | 1 |
| 27 | 1 |
| 28 | 1 |
| 29 | 0 |

| | |
|---|---|
| 30 | 1 |
| 31 | 1 |
| 32 | 1 |
| 33 | 1 |
| 34 | 1 |
| 35 | 0 |
| 36 | 1 |
| 37 | 0 |
| 38 | 1 |
| 39 | 1 |
| 40 | 0 |
| 41 | 0 |
| 42 | 0 |
| 43 | 1 |
| 44 | 1 |
| 45 | 1 |
| 46 | 1 |
| 47 | 1 |
| 48 | N/A |
| 49 | 1 |
| 50 | 1 |
| 51 | 1 |
| 52 | 1 |
| 53 | 1 |
| 54 | 0 |
| 55 | 1 |

**Supplementary Table S6. Consumer demographics as percent of overall study**

| Age | | | Gender | | |
|---|---|---|---|---|---|
| 18 to 24 | 25 to 34 | 35 to 47 | Female | Male | Non-binary |
| 59.3% | 35.2% | 5.6% | 47.3% | 47.3% | 5.5% |

| Highest Level of Education | | | | | |
|---|---|---|---|---|---|
| High School | Some College | Technical School | Bachelor’s | Master’s | Doctorate |
| 7.3% | 32.7% | 0.0% | 45.5% | 7.3% | 5.5% |

| Annual Household Income | | | | | | | | |
|---|---|---|---|---|---|---|---|---|
| Under $20,000 | $20,000 - $39,999 | $40,000 - $59,999 | $60,000 - $79,999 | $80,000 - $99,999 | $100,000 - $119,999 | $120,000 - $139,999 | $140,000 - $159,999 | $160,000 and over |
| 13.5% | 11.5% | 26.9% | 5.8% | 5.8% | 7.7% | 1.9% | 3.8% | 23.1% |

| **Would you be willing to buy cultivated meat if it has the same price as conventional meat?** | **Would you be willing to pay 10% more for cultivated meat?*** | **Would you be willing to buy cultivated meat at a 10% discount?**** |
|---|---|---|
| Yes: 98.2% | Yes: 81.5% | Yes: 100.0% |
| * = out of participants who would buy cultivated meat at the same price as conventional meat.<br>** = out of participants who would **not** buy cultivated meat at the same price as conventional meat. | | |
